# Patient experiences of shared decision-making following a displaced collarbone injury: A qualitative interview study

**DOI:** 10.1177/02692155251355440

**Published:** 2025-06-30

**Authors:** Natasha Maher, Maria Clare Moffatt, Felicity Astin, Chris Littlewood

**Affiliations:** 1 Calderdale Royal Hospital, Calderdale and Huddersfield NHS Foundation Trust, Halifax, UK; 2 School of Allied Health Professions and Nursing, 4591 University of Liverpool, Institute of Population Health, Liverpool, UK; 3 School of Health, Wellbeing and Social Care, Faculty of Wellbeing, Education and Language Studies, 5488 The Open University, Milton Keynes, UK; 4 7046 University of Salford, Salford, UK

**Keywords:** Interview, collarbone, shared decision-making, qualitative, patient-centred care

## Abstract

**Objective:**

To explore the patient experience of shared decision-making following a displaced collarbone injury, focusing on how patients understand their injury and how this influences decisions.

**Design:**

Descriptive qualitative study design using individual semi-structured interviews.

**Setting:**

Participants recruited from three United Kingdom National Health Service hospitals.

**Participants:**

Patients with a displaced collarbone injury were interviewed about their experiences of shared decision-making.

**Main measures:**

Interviews were audio-recorded, transcribed verbatim and analysed using inductive thematic analysis.

**Results:**

Three themes emerged: (1) Understanding of the injury, (2) Factors influencing treatment decision and (3) Experience of shared decision-making. Patients’ interpretation of their injury, including the language used by clinicians, shaped their understanding and decisions. Factors such as previous injuries, employment, clinician advice and expectations also influenced treatment choices. Some patients described uncertainty during decision-making conversations and felt unsupported in choosing the option that best suited them. Others felt steered towards specific treatments without fully grasping their implications.

**Conclusion:**

This is the first qualitative interview study exploring patients’ perspectives of shared decision-making following a displaced collarbone injury. While patients considered several factors when deciding between treatment options, many described limited involvement in decision-making and felt directed towards clinician-preferred treatments without fully understanding the implications. This highlights inconsistency in the implementation of shared decision-making in practice. Despite the United Kingdom National Health Service emphasis on shared decision-making, further efforts are needed to ensure that patients are actively supported in making informed, preference-sensitive decisions, in line with the goals of personalised care.

## Introduction

Patient-centred care is a central ethos of the United Kingdom National Health Service Long Term Plan.^1^ Shared decision-making is a key component of patient-centred care which aims to support patients to make decisions that are right for them.^2^ Shared decision-making principles also require that people are aware of relevant choices and consider their personal views and preferences when determining their best healthcare option.^3^ However, it is apparent that clinician-led decisions about treatment still prevail in many areas of the United Kingdom National Health Service.^4^ Where research evidence does not indicate one superior treatment for a patient, shared decision-making is important to help people compare options and make more informed choices.^3^

Displaced collarbone injuries are one clinical presentation where the research evidence is equivocal.^5,6^ Management of displaced collarbone injuries could include wait-and-see, physiotherapy and/or surgery to stabilise the displaced collarbone.^7^ However, there is uncertainty about the optimal treatment of these injuries and often the treatment pathway can be dictated by the professional background and preference of the treating clinician.^8^ Shared decision-making relies not only on presenting options but also on the patient's capacity to understand their condition and weigh the benefits and risks of each treatment. In musculoskeletal injuries, such as displaced collarbone injuries, patients’ understanding of the nature and severity of their injury can shape how they interpret clinical advice, perceive urgency and engage in decision-making. Misunderstandings or inconsistent communication from clinicians may hinder patients’ ability to participate meaningfully in this process.^9^

Preference-sensitive decisions, where both healthcare professionals and patients agree that uncertainty about the optimal treatment exists, are an ideal basis for shared decision-making.^9^ Hence, due to a lack of high-quality evidence relating to optimal treatments for displaced collarbone injuries, there is a platform from which shared decision-making can usefully evolve. However, no previous research has described the patient experience of sustaining a displaced collarbone injury and how shared decision-making is used within the treatment pathway.

Hence, the aim of the study was to understand the patients’ perspective of shared decision-making in a United Kingdom National Health Service setting following displaced collarbone injuries including how patients understand their injury and how this understanding influences decision-making.

## Method

A descriptive qualitative research study with semi-structured interviews was undertaken to explore participants’ experiences of shared decision-making within their treatment across three United Kingdom National Health Service hospitals. This study is reported in line with the COnsolidated criteria for REporting Qualitative research checklist.^10^ A qualitative design was chosen over quantitative methods in order to explore the richness and depth of the participants’ experiences which would not be possible with a quantitative method such as a survey or service evaluation.^11^ In health research, qualitative methods are considered as the most humanistic and person-centred way of discovering and uncovering thoughts and actions of human beings.^11^ A sample size of approximately 10 was estimated a priori based on practical considerations and methodological precedent, with final numbers determined by thematic saturation.

The patient and public involvement group supported the design of the interview study by helping to develop the interview topic guide (Supplementary File 1) and with the patient-facing information used during the recruitment process. Ethical approval was obtained on the 30^th^ of November 2023 from Wales Research Ethics Committee 5, Bangor, and the United Kingdom Health Research Authority (ref: 23/WA/0278).

### Recruitment

A convenience sample of 10 participants with a displaced collarbone injury, (excluding fractures) where surgical and non-surgical treatment options are viable, was recruited from three United Kingdom National Health Service hospitals. We opened recruitment at four United Kingdom National Health Service hospital sites, but one site was unable to recruit a patient within the allotted time frame. For the purpose of this unfunded study, we limited our eligibility criteria to participants who could communicate in English.

Participants were eligible for recruitment to the study if, during their usual United Kingdom National Health Service treatment, they fulfilled the following clinical criteria:

Any adult (over the age of 18) with a displaced collarbone injury (excluding fractures).

Had a displaced collarbone injury confirmed via x-ray.

The patient had attended either Accident and Emergency services, Musculoskeletal services, physiotherapy services or an orthopaedic service for management of this injury.

Could communicate in English.

Had the capacity to consent.

Participants were only included if, at the time of clinical presentation, there was clinical equipoise regarding surgical versus non-surgical management, meaning more than one treatment option was considered reasonable by the treating clinician.

When a patient met the eligibility criteria, the local United Kingdom National Health Service clinician responsible for their care introduced the study, provided the patient with a participant information sheet, and sought consent from the lead researcher (NM) to contact them to discuss the study further. The consent to contact form was completed and stored locally in the investigator site file and an electronic copy scanned and sent to NM. The contact information of eligible potential participants was also shared with NM via a United Kingdom National Health Service mail (either nhs.net or nhs.uk) account.

The patient was given the participant information sheet by the local clinician and then contacted via telephone by NM following this. Participants were asked if they had any questions and whether they would be interested in participating. If they did not want to participate, they were thanked for their time and the call ended. If interested and willing, the initial consent was audio-recorded, and the participant filled out the ‘Patient Consent Form’ and posted back to NM using the stamped address envelope provided. Initial verbal consent meant that interviews could be scheduled and undertaken, if appropriate, at the time of this initial conversation. Participants were made aware that they could withdraw their data up until the point it was included in the final data analysis. Consent to publish was obtained.

### Data collection

Participants were offered an interview using Microsoft Teams or via telephone according to their preference. Prior to each interview, NM introduced herself as a mixed white and Asian, female physiotherapist with a post-graduate qualification in advanced physiotherapy, who has undertaken formal qualitative research training and is the lead researcher for the study. Semi-structured interviews were conducted using a topic guide that was developed by the research team and Patient and Public Involvement group and informed by relevant literature. Interviews were recorded, transcribed and verbatim and anonymised by NM. Transcripts were not returned to participants. They were then shared with the wider research team to support critical discussion and any refinement of the interview questions, if needed, to ensure the objectives of the study were being met.

We initially aimed to recruit a convenience sample of approximately 10 participants, based on prior qualitative research in similar clinical settings and the scope of our research question. However, we used an iterative approach to data collection and analysis, drawing upon the principle of thematic saturation.^12^ As interviews progressed and themes began to recur, we continued recruitment until no new themes were emerging from the data, at which point thematic saturation was deemed to have been achieved and recruitment ceased.^12^

### Data analysis

Recruitment, data collection and analysis occurred sequentially to enable collected data to inform subsequent interviews and to allow the researchers to cease recruitment once thematic saturation was achieved. MM and NM independently undertook initial coding on a line-by-line basis using ‘open coding’ to allow multiple codes to be applied to single sections of data. Transcripts were reviewed several times to assist with data immersion. CL and FA cross-checked transcripts and coding, with all disagreements resolved through repeated discussions. Documented themes were critically discussed with all authors (MM, CL and FA) following five interviews and following a further five interviews to support rigour, further develop the emerging analysis and to ensure themes characterised participants’ reported experiences and views. The coding framework is presented in Supplementary File 2.

## Results

The study was discussed with 14 potential participants, 4 declined and 10 consented to participate in the study. Four participants were recruited from United Kingdom National Health Service hospital 1, four from United Kingdom National Health Service hospital 2 and two from United Kingdom National Health Service hospital 3. The interviews ranged from 14 to 35 min (mean 21 min). All participants were male; this is reflective of the typical demographic of people who experience a displaced collarbone injury.^7^ Participant demographics are reported in Table 1.

**Table 1.** Participant demographic information.

Study ID No.	Gender	Ethnicity	Employment status	Profession of consulting clinician	Type of interview
PT-101	Male	White-Arabic	Employed full time	Orthopaedic Consultant and physio (sought private consultation prior to United Kingdom National Health Service appointment)	Phone
PT-102	Male	White-British	Self-employed	Orthopaedic Consultant	Phone
PT-103	Male	White-British	Employed full time	Physio	Microsoft Teams
PT-104	Male	White-British	Employed full time	Physio	Phone
PT-105	Male	White-British	Employed full time	Orthopaedic Consultant	Phone
PT-106	Male	White-British	Retired	Physio	Phone
PT-107	Male	White-British	Employed full time	Participant unsure of clinician profession	Phone
PT-108	Male	White-British	Employed full time	Physio	Phone
PT-109	Male	White-British	Employed full time	Orthopaedic Consultant	Phone
PT-110	Male	White-British	Employed full time	Physio	Phone

Participants’ experiences were characterised by three themes.

### Theme 1: Understanding of the injury

Participants had varying levels of understanding and interpretation of their collarbone injury, with inconsistent terminology used in consultations. Different terms, such as dislocation, separation and displacement, were used to describe the injury. This language influenced participants’ perceptions of prognosis and whether they felt surgery might be required. For example, one participant expressed frustration when told their injury was ‘displaced’ rather than ‘dislocated’, believing the latter would heal more quickly:

Just after I heard it was displaced, I…wished it was dislocated so he could just put it back in the right place… because it's been displaced it has to be kind of a slow change which has been infuriating [PT 107].

Another participant immediately felt surgery was necessary when told that their

‘bones had moved… it's not going to go back’ [PT 108].

Only two participants reported that they felt they had a clear understanding of their injury prior to their consultations. PT 101, with a background in personal training, had researched the injury and made a decision to pursue surgery based on this:

I used PubMed and Google Scholar and I just looked at current research around ACJ separations [PT 101].

Similarly, PT 110, who had undergone a previous collarbone stabilisation, was familiar with the surgical process. For those without this prior experience, less certainty regarding treatment options was apparent.

The Rockwood classification system, commonly used to describe the severity of displaced collarbone injuries, was sometimes a source of confusion. Participants were told conflicting grades by different clinicians, which led to misunderstanding. For instance, one participant observed a poster indicating stages 1–3 of injury severity but was later told by a clinician that their injury was a ‘stage 5’, which they found concerning:

There was a poster in the minor injuries section… he said it was stage 5, I thought…that sounds like it's really really bad, I need to get something done here. That was a little bit confusing [PT 103].

Another participant was given differing grades by two clinicians:

Clinician X graded it a grade 5, and Consultant Y graded it a grade 3 [PT 101].

There also appeared to be some variation in how injuries were explained based on the profession of the clinician. Participants who were seen by orthopaedic consultants were more likely to receive medically framed explanations or to associate their diagnosis with a surgical pathway. In contrast, physiotherapists often provided more general or function-oriented descriptions, which sometimes left participants unsure about the severity of the injury or the need for surgery. One participant who consulted both a physiotherapist and a consultant observed a noticeable shift in language and framing, which impacted his understanding of the options available. These inconsistencies in understanding and communication significantly affected participants’ experiences of shared decision-making, highlighting the need for clearer, more consistent explanations during consultations as well as checking patient understanding through dialogue rather than monologue.

### Theme 2: Factors influencing treatment decision

Several factors influenced participants’ treatment decisions, including occupational considerations. For some, returning to work quickly was a key factor, either pushing them towards surgery, based on the assumption that was the superior treatment option in this regard, or discouraging it. For example, one participant emphasised that his ability to work was tied to his physical health:

It's my livelihood… If I can't use my body in this current field, then I can't make money [PT 101].

Another, a welder, felt he needed surgery to regain full use of his arm:

It's not like I need the surgery…if I was still at school, it would be grand because I’d just get the surgery but because I’m at work… I need my right arm [PT 109].

The timing of surgery was also a major consideration for many participants. Eight reported that the non-urgent nature of the surgical option influenced their decision to initially opt for non-surgical management:

If you'd have told me it was just then and there, I’d have probably just took the surgery but because I can get it at any point, it's like what's the rush? [PT 109].

Similarly, one participant was relieved to learn there was no immediate need for surgery:

I don’t have to decide that now [PT 106].

Another participant mentioned that the non-time-sensitive nature of the injury helped frame the decision-making process:

She said it's not time sensitive, she painted the picture for me and sometimes it does get better and sometimes it doesn’t [PT 104].

Previous experiences with surgery also played a role. One participant, who had previously undergone collarbone stabilisation surgery on the other side, was reluctant to pursue surgery again:

Firstly, I dreaded the prospect of having to have surgery again [PT 110].

Another shared a negative experience with knee surgery where he felt he was encouraged to proceed with surgery which and had not had a successful outcome.

This affected his decision-making following this collarbone injury. Fear or poor outcome after previous surgery made participants hesitant to rush into any further surgeries without careful consideration:

I sort of rushed into getting an operation on my knee… I didn’t want to rush into an operation anyway [PT 104].

Personal attitudes towards surgery also influenced decisions. One participant viewed surgery as the most reliable solution to avoid complications:

I just think surgery is doing the, it's the most sure-fire way to get back to where I was without any issues [PT 101].

Conversely, another participant, who had a negative preconception of doctors and hospitals, leaned towards non-surgical treatment:

I’ve never had an operation, but I don’t like the sound of it. I’m not a massive fan of doctors or hospitals [PT 105].

Six participants were presented with multiple treatment options, and for some, this had a positive effect on their experience. However, for others, it felt overwhelming. One participant appreciated having clear options laid out:

Highlighting both sides was really useful [PT 101].

Another reflected on the weight of the decision after being given options:

I went away thinking it's going to be on me now, what do I want?… I have to decide something [PT 103].

A third participant felt the options were presented clearly, but ultimately left with the responsibility of deciding:

The options were clearly laid out for me… the one thing I wanted to know was do I need an operation or not [PT 106].

The clinician's profession also influenced how treatment recommendations were delivered. Participants who saw orthopaedic consultants more frequently reported feeling directed towards surgery, or that the option was framed as the preferred clinical route. In contrast, those seen by physiotherapists described a more exploratory or balanced approach, sometimes involving broader discussions about recovery and self-management. This variation affected not only how participants perceived their options but also the level of decisional pressure or ownership they felt during the process. Additionally, participants did not report receiving written materials such as leaflets or decision aids during consultations. Most relied on verbal explanations, gestures or their own online research. This lack of standardised information may have contributed to confusion and limited opportunities to support informed decision-making.

### Theme 3: Experience of shared decision-making

Participants’ experiences of shared decision-making were inconsistent. Some felt positive about their consultations despite being guided towards a particular treatment. These participants were content to defer to the clinician's expertise and suggested that they did not feel the need to be fully involved in the decision-making process. For example, one participant described being led towards treatment by a physiotherapist:

I didn’t walk away thinking I really feel like I want to go for the surgery and they kind of made me go for the physio [PT 101].

Another reflected on feeling hopeful about the consultation outcome despite not making a firm decision themselves:

I think we were both pretty happy with… I was pretty hopeful [PT 104].

A third participant expressed trust in the clinician's guidance:

I’m always trusting in the experts really… I think sometimes when people give me advice, I sort of process it and think well yeah that sounds right to me [PT 110].

In contrast, several participants reported decisional uncertainty and a perceived lack of support to make an informed treatment decision, leaving them dissatisfied. One participant noted the pressure of having to make a decision alone:

I went away thinking it's going to be on me now, what do I want?… I have to decide something [PT 103].

Another expressed a desire for more shared responsibility:

I would have liked more of a shared responsibility [PT 104].

Some participants felt they were subtly encouraged towards one treatment without fully understanding the implications. Despite being presented with multiple options, the focus on what mattered most to the patient, a core element of shared decision-making, was lacking. For example, one participant noted that surgery was mentioned but not truly offered as a choice:

Surgery was put on the table but it wasn’t really offered… it wasn’t even like I was given surgery vs physio [PT 107].

Another recalled being warned about the potential downsides of surgery:

If you want to go through surgery… it will probably cause more problems than answers [PT 105].

One participant felt that surgery was not seriously considered at all:

I don’t think surgery was mentioned at all [PT 108].

These experiences suggest that while the intention to facilitate shared decision-making is present, their core components such as patient involvement, the consideration of individual preferences, along with time for deliberation, are not consistently integrated into clinical consultations.

## Discussion

To our knowledge, this is the first qualitative study of patients’ experiences of shared decision-making in relation to their displaced collarbone injury. The key findings suggest there is a lack of consistency in the way shared decision-making is perceived and understood by patients as a process to enable patients to make a decision that is right for them. Inconsistencies were also apparent in the language and terminology used to describe the participants’ injury.

It has long been recognised that healthcare should be patient-centred and that patients should be involved in decisions about their care.^13^ This is particularly important when multiple treatment options are available to address patients’ needs, especially when these options differ in ways that matter most to them.^13^ Most models of shared decision-making aim to address three key steps in the decision-making process: (1) creating choice awareness, (2) discussing relevant treatment options and (3) discussing patient preferences.^13^ To date, most shared decision-making implementation research has mainly focused on the second and third steps of shared decision-making and on making the final treatment decision.^13^ However, ‘creating choice awareness’ involves acknowledging that the patients’ situation is changeable and that there might be more than one way to address their situation.^14^ Adequate choice awareness is therefore a vital, but often missed, component of shared decision-making.^13^

While shared decision-making is widely promoted, our findings suggest it may not suit all patients equally. Some participants valued being involved, while others felt burdened by the responsibility, particularly when uncertain about their options. One participant noted, ‘It's on me now’, reflecting this emotional weight. These findings raise the question of whether patients should first be asked how much involvement they want in decisions. In preference-sensitive decisions, where multiple reasonable options exist and patient values are central, tailoring the shared decision-making process to individual preferences for involvement may better support patient needs.^15^

It was apparent that several factors appeared to influence the participants’ treatment decisions. These factors included their occupation, the time-sensitivity of the operation, the patients’ previous healthcare experiences and the experience of the clinician. Waddell and colleagues supported this theme through their description of several patient-related factors.^16^ Patients who are well informed prior to the shared decision-making discussion, report feeling able to engage in shared decision-making conversations with their clinician.^16^ However, respecting patients’ choice to be involved in decision-making (or not) should occur in a context in which they understand what involvement means, recognising that there are not necessarily right or wrong decisions in situations of uncertainty about the clinical effectiveness of different treatment approaches, and appreciate that they have a valuable contribution to bring to a healthcare encounter.^17^ Paucity of knowledge of the risks and benefits of different treatment options was evident in our findings. This is documented as a barrier for both clinicians who are trying to explain the different options and for patients trying to understand them.^16^

Patients with prior injury experience or who accessed academic sources like PubMed appeared more confident in their decisions. Others, relying only on verbal explanations, expressed confusion or uncertainty. This highlights the potential role of decision aids; tools that present treatment options, benefits and risks to support informed decision-making. Evidence shows decision aids can improve patient knowledge and reduce decisional conflict, especially in musculoskeletal care.^15^ Future research should explore their development for acute orthopaedic injuries such as displaced collarbone injuries.

In the context of shared decision-making, solely presenting evidence-based information about the effectiveness of different treatment options to patients so that they can decide the best treatment option in isolation is considered insufficient, as it often leads to an unrealistic responsibility for patients.^18^ In some cases, like participants in this study, encountering ‘it's your decision’ or ‘it's up to you’ may cause patients to feel a sense of abandonment, rather than feeling empowered or cared for.^18^ However, the patients’ role in the process should not be limited to just voicing their preferences, only for the clinician to decide what needs to be done either.^18^ It is useful to consider that patient preferences usually evolve within the deliberation process, while considering the situation and the options available. Thus, they rarely exist ahead of the consultation.

This study highlights key clinical and research implications. Clinically, clearer and more consistent communication is needed to support patient understanding and meaningful shared decision-making. Not all patients want the same level of involvement, reinforcing the need for a flexible, preference-sensitive approach. From a research perspective, there is a clear need for evidence-based decision aids tailored to musculoskeletal injuries, as well as further investigation into how clinician role, communication style and patient preference interact to shape decision-making outcomes.

This qualitative interview study gives a voice to adults experiencing a displaced collarbone injury having been treated in a United Kingdom National Health Service hospital. The reflexive thematic analysis approach used, allowed rich engagement with the data to produce detailed accounts of each participant's experiences. Participants were given the choice of a Microsoft Teams or audio-recorded phone interview depending on their preferences. Our decision not to offer a face-to-face interview could be a potential limitation due to possible difficulties in building a rapport and observing non-verbal cues.^19^ However, literature suggests that telephone interviews can generate data that is comparable to in-person interviews,^20^ and the relative anonymity perceived during telephone interviews may inspire participants to disclose more in-depth opinions.^21^ Our decision to use telephone interviews is, therefore, not a threat to the study's rigour.

Our study did not include any female participants, and only one participant was from a non-White-British background. It is important to acknowledge that this sample may have differed from the broader population (e.g., certain characteristics that may have meant their experiences of shared decision-making differed). However, it is widely accepted that men have a 5–10 times higher risk of suffering a displaced collarbone injury in comparison to women,^7^ which is reflected in our sample. We also intended to have participants for four United Kingdom National Health Service hospitals; however, one of the hospitals was unable to recruit a participant within the allocated time frame. Due to the nature of this small sample, these findings may not represent the experiences of those treated in other United Kingdom National Health Service hospitals or outside of the United Kingdom National Health Service setting.

While patients considered a range of factors when weighing treatment options, many described limited involvement in decision-making and felt that decisions were primarily clinician-led. This study reveals a clear inconsistency in how shared decision-making is supported in practice. Despite the United Kingdom National Health Service commitment to shared decision-making, these findings suggest that it is not yet embedded as standard practice. Further efforts are needed to ensure patients are meaningfully involved in decisions and supported with consistent, understandable information, a necessary step towards achieving truly personalised care.

**Clinical messages**

- Patients often lack clear, consistent explanations about their injury and treatment options.
- Shared decision-making is inconsistently applied and can leave patients feeling unsupported or overwhelmed.
- Clinicians should actively assess patients’ decision-making preferences and use structured tools to support informed, personalised care.

## Supplemental Material

sj-docx-1-cre-10.1177_02692155251355440 - Supplemental material for Patient experiences of shared decision-making following a displaced collarbone injury: A qualitative interview study Supplemental material, sj-docx-1-cre-10.1177_02692155251355440 for Patient experiences of shared decision-making following a displaced collarbone injury: A qualitative interview study by Natasha Maher, Maria Clare Moffatt, Felicity Astin and Chris Littlewood in Clinical Rehabilitation
