# Supplementary material for: Patient experiences of shared decision-making following a displaced collarbone injury: A qualitative interview study
Source: Clin Rehabil. 2025 Jun 30;39(8):1105–15. doi: 10.1177/02692155251355440 (PMC12290225; doi:10.1177/02692155251355440)
Supplement: sj-docx-1-cre-10.1177_02692155251355440 - Supplemental material for Patient experiences of shared decision-making following a displaced collarbone injury: A qualitative interview study [file sj-docx-1-cre-10.1177_02692155251355440.docx]

Supplementary file 2. Coding Framework

| Codes | Participants | Relevant Quotes | Code description | Theme |
| --- | --- | --- | --- | --- |
| 1. Understanding of the injury | PT101, PT102, PT103, PT 106, 107, PT 108, PT 109 | - ‘dislocated collarbone’ 101 - ‘ligaments have snapped’ 101 - ‘ACJ separation’ 102’, - ‘I thought I’d broke my collarbone… he was like, you haven’t broken your collarbone, you’ve actually got an ACJ separation’ 101 - ‘shoulder dislocated and collarbone had popped, torn your ACJ, Grade 3 ACJ’ 109, - ‘ it was not dislocated it was displaced’ 107 - ‘Id never heard of a displacement before’ 107 - ‘The ligaments stretching and collarbone not snapping and how the impact might move my bones’ 108 - ‘It would probably never go back to normal’ 107 - ‘It’s probably not going to heal as quick’ 108 - ‘How was the condition explained to you… it wasn’t 105’ - ‘My collarbone. They told me I rotated it whilst still staying in the joint’. 107 - ‘its not like I need the surgery, and if the surgery, if I was still at school it would be grand because I’d just get the surgery but because I’m at work, especially at my work because works about 8 weeks at least because I’m a welder, I need my right arm’ 109 - ‘They did grade it differently. Clinician X graded it a grade 5, and Consultant Y graded it a grade 3’ 101 - ‘I mean they said it was displaced and I asked them because Id never heard of it and I was like what does that mean and they basically showed the joint with their hands and showed me that displaced means it never left the joint it just rotated’ 107 - ‘She thought it was a grade 5’ 101 - ‘I dint know what I’d done. They did Xray it in A and E and said you haven’t broken anything. So that were the main concern that I’d broken something. But what got me is that if I had have broken anything, then why couldn’t I move my arm?’ 105 - ‘Did you understand anything about the condition before you went to A and E?-‘nothing at all to be honest’ 108 - ‘I used PubMed and google scholar and I just looked at current research around ACJ separations’ 101 - ‘I’ll be honest I was just after I heard it was displaced, I probably just wished it was dislocated so he could just out it back in the right place. Because I don’t know whether it just an illusion, but I thought if it was dislocated maybe I’d just if I could get it back in the right place and then it would be fine after a month or two but because its been displaced it has to be kind of a slow change which has been infuriating because I like being an active person’ 107 - ‘T-Ok so was that something you were expecting to talk about… surgery?’ - ‘P-Well when he said my bones had moved, obviously I thought, but he said its only slight, its not going to go back I didn’t know if there was surgery to put that back or whatever’ 108 - ‘ I’ve got physio people telling me, Oh well it could happen to anyone that. Then until the actual ultrasound lady says, no you’ve got something not quite right then I dint know owt about it’ 105 - ‘Its meant to connect to the rest of the shoulder and that’s what had broken. So I was concerned about do they repair themselves because with bones, you put them back together and they knit and they’ll be back together again. I was worried about if these ligaments are broken, can they be repaired’ 103 - ‘When I had the first one done, it was sort of classed as a Grade 5, there wasn’t kind of any messing around. It was you know surgery is the best solution so that’s what we went for’ 110 | -Patients understanding and interpretation of the injury, use of terminology | Theme 1- Understanding of the Injury |
| 1. Patient expectations | PT101,102 103, 104, 108 | - ‘For my injury personally, I don’t think it was bad enough for anything more than physiotherapy’ 109 - ‘I just think surgery is doing the, it’s the most sure fire way to get back to where I was without any issues (PT101 in his words not clinicians)’ - ‘I wasn’t expecting it to be like that’ 101 | Patients’ expectations of the injury, the consultation, their outcome | Theme 2 – Factors influencing treatment decision |
| 1. Given multiple treatment options to choose from | PT101, 102, 103, 104 106 109 | - ‘With my grade of injury you can go one of 2 ways, typically you can go for a surgery or you can go for a more conservative approach’ 101. - ‘So, we talked about the pros and cons of having an operation and also that I don’t have to decide that now’ 106 - ‘I think that the options were clearly laid out for me’ 106 - ‘I think that the options were clearly laid out for me, I wasn’t precious one way or the other’ 106 - ‘I think I got what I got when I needed it’ 106 - ‘’they ultimately detailed the options and then gave the option to discuss… so what do you want to do about this? ‘101 - ‘the one thing I wanted to know was do I need an operation or not. Her view was its up to you pretty much’ 106 - ‘I definitely felt involved with the decision yes because they ultimately detailed the options and then they gave the option to me to discuss so what do you want to do about this’ 101 - ‘Highlighting both sides was really useful’ 101 - ‘And so you felt that the way she explained it to you made sense to you and was tailored to what you required in that consultation. P-Absolutely’ 106 - ‘Yeah so I’m still in that situation now really because the physio, was saying really its kind of cosmetic, you can either just leave it as it is or you can… This wasn’t her opinion this was her saying what she thought the Consultant would say’ 103 | Given more than one option for treatment, explained what would happen with the different options | Theme 2 – Factors influencing treatment decision |
| 1. Factors influencing treatment decision | PT101, 102, 103, 104, 105, 106, 108, 109, 110 | - ‘I can get it any time so if I have any hassle with it in the future I can always come back’ 109 - ‘100%...if you’d have told me it was just then and there, Id have probably just took the surgery but because I can get it at any point’ 109 - ‘Its my livelihood… If I cant use my body in this current field then I cant make money’ 101 - ‘For me, personally, it was sort of just what I had to do [conservative] because I would have to go back to work’ 108 - ‘We discussed the risks {work]… its probably not going to heal as quick’ 108 - ‘I would be able to just ring straight up and they would get straight in there and get another appointment’ 108 - ‘I…sort of rushed into getting an operation on my knee… I didn’t want to rush into an operation anyway’ 104 - ‘I don’t like the sound of it [surgery] 105’ - ‘I’ve got a friend who did the same thing, I recall his discussions were about do we operate or do we not operate’ 106 - ‘I didn’t think I was going to get as good of an explanation as I received’ 108 - ‘the other shoulder… its serving me really well… so you know I was pleased with the outcome of the treatment’ (110- had previous ACJ injury on the other side) - ‘She [partner] didn’t want me to go through the surgery’ 110 - ‘he suggested, was if you’re in 2 minds about it. Live with it and see how you get on. I said, well yeah that’s the best option’ 102 - ‘There was a poster in the minor injuries section at [UK NHS Trust], and they said it went stage 1,2 and 3 and it showed the different things that stage 1, 2 and 3 was. I went to see the guy in minor injuries and he said it was stage 5, I thought that’s a bit weird, that sounds like its really really bad, I need to get something done here’ 103 - ‘I didn’t get pushed into it but sort of rushed into getting an operation but my knee’s still buggered so I kind of just didn’t want to, I didn’t want to rush into an operation anyway’ 104 - ‘I’ve never had an operation but I don’t like the sound of it. I’m not a massive fan of doctors or hospitals’ 105 - ‘if I go down the route of having an operation then I’ve got to recover from having an operation’ 106 - ‘Its like what’s the rush? Why rush into something so soon?’ 109[in reference to surgery] - ‘Normally they would have operated but insurance in the UK doesn’t cover it’ 106 - ‘I’m always trusting in the experts really’ 110 - ‘she said its not time sensitive, she painted the picture for me and sometimes it does get better and sometimes it doesn’t’ 104 | Cosmetic, work, not a time sensitive decision, past experience, friends and family past experience, external factors) | Theme 2- Factors influencing treatment decision |
| 1. Perceived lack of support to make treatment decision | PT101, 103, 104, 109 | - ‘Would have liked more of a shared responsibility 104’ - ‘ I feel like it was my idea completely really about the decision about the surgery’ 109’ - ‘The one thing I wanted to know was do I need an operation or not. Her view was its up to you pretty much??’ - ‘They basically passed the choice to me and said you know basically I’ve given you the options what do you want to do? 101 - ‘I went away thinking its going to be on me now, what do I want?... I have to decide something’ 103 - ‘I left walking away thinking lets see how it goes?’ 104 - ‘what if I can never move it again?105’, well I didn’t know what were wrong with it?’ 105 - ‘I didn’t know the choice would be mine. I didn’t think that would be up to me. I thought that would be up to the Consultant themselves’ 101 - ‘given your situation you can go either route so what would you like to do?’ 101 - ‘I think it’s a cosmetic type problem rather than a problem that needs attention and if its that its kind of me to decide and I’m not sure I’ve got all the information I need to make that decision’ 103 - ‘So I feel as though I’m lacking that information’ 103 - ‘here I seem to be expected to make a decision and its not a clinical decision because if it was I’m sure she would have made it and would have said, no we need to operate on it very quickly because its serious. But because it just sort of, I’m not sure if cosmetics the right word but because its non-threatening in any way then it seems, oh we can so something about it certainly and we can probably do it on the NHS but do you want it doing so I’m thinking I don’t know’ 103 - ‘but I feel like I’m going to have to live with my decision regardless of what happens’ 103 - ‘It will be my problem then wont it. If I make the wrong decision I’ll only have myself to blame so I don’t plan on doing it’ 103 - ‘I wasn’t precious one way or the other. The decisions were mine to make and I was given a lot of information. I think I’ll be able to make an informed decision’ 106 - ‘I mean initially but I don’t know if she’s not allowed to give her opinion on whether I should have surgery or not but I did ask her, I was like what should I do? She was like its up to you. She obviously doesn’t have the, I don’t know she’s not allowed to obviously. So that was tricky, it was sort of left in mind and I left walking away thinking lets see how it goes’ 104 | Feeling like they had to make the decision alone, may have been given options but interpreted this as them having to make decision themselves | Theme 3- Experience of shared decision making |
| 1. Encouraged to go for a particular option | PT 105, PT 108, PT 109, 110 | - ‘If you want to go through surgery…you know if you did that it will probably cause more problems than answers’ 105 - ‘If it had been Forrest Gump I would have been led by him’ 105 - ‘I didn’t really get given many options’ 108 - ‘It wasn’t really a decision I was more led by the Consultant’ 105 - ‘He told me, to be fair with the injury, I can live with it, its not like I need the surgery’ 109 - ‘I don’t think surgery was mentioned at all’ 108 - ‘I’m quite easily led and I think sometimes when people give me advice, you know I sometimes accept it…I sort of process it and sort of think well yeah that sounds right to me’ 110 - ‘do you think it was sort of evenly balanced offered to you? P-Oh no definitely not. He was definitely saying don’t do it’ 105 - ‘do you feel that you were involved with that decision making process, P-No not at all. Definitely not. I was led by the lad. He’s the professional’ 105 - ‘they told me we could do surgery, but they would rather avoid it and see how it goes and then if it stayed really bad then they would operate on it, but they said they would rather avoid it because it should deal with itself’ 107 - ‘Yeah as I said the alternate… surgery was put on the table but it wasn’t really offered if that makes sense so they said you can so surgery but we’ll only do it if it stays bad so it wasn’t even like I was given surgery vs physio’ 107 - ‘T-Did anyone discuss surgery with you? P-Erm no. I don’t think surgery was mentioned at all’ 108 - ‘it seemed to be leaning more heavily towards a conservative approach rather than a surgical approach’ 110 - ‘I felt like I was kind of being encouraged almost to agree, I mean I’m the sort of person who was given this information, I trusted them and the way they put it across I absolutely agreed with them’ 110 - ‘Surgery wise they just told me that they could put it back in place with surgery but then there’s the dangers of actually going into a surgery but if you’re asking me how they explained what the surgery process was I was just told that they put it back in place. They didn’t really tell me what the surgery was’ 107 - ‘It could happen to anyone that’ 105 - ‘Surgery wise they just told me that they could put it back in place with surgery but then there’s the dangers of actually going into a surgery but if you’re asking me how they explained what the surgery process was I was just told that they put it back in place. They didn’t really tell me what the surgery was’ 107 - ‘He said he worked with people and they’ve lived their lives no hassle and just got on with it’ 109 - ‘I don’t have any choice’ 104 | Encouraged into making one particular choice | Theme 3-Experience of shared decision making |
| 1. Patient experiencing decisional uncertainty | PT 103, 105, 108, 109 | - ‘I’m happy to make the decision, If I’ve got all the information to make the decision’ 103 - ‘Here I seem to be expected to make a decision and it’s not a clinical decision because if it was I’m sure she would have made it and would have said, no we need to operate on it very quickly because its serious. But because it just sort of, I’m not sure if cosmetics the right word but because its non-threatening in any way then it seems, oh we can do something about it certainly and we can probably do it on the NHS but do you want it doing so I’m thinking I don’t know’ 103 - ‘Yeah as I said the alternate… surgery was put on the table but it wasn’t really offered if that makes sense so they said you can do surgery but we’ll only do it if it stays bad so it wasn’t even like I was given surgery vs physio’ 107 - ‘It didn’t feel 100% like the right decision’ 110 - ‘if I didn’t have the operation straight away so that was what I was worried about with my shoulder’ 104 [worried about going ahead with the operation] - ‘I guess if you operate, is that a permanent fix? Or is it something that you don’t have to worry about it’ 103 | Decisional regret, decisional conflict, uncertainty, not feeing like they had all the information to make the decision | Theme 3- Experience of shared decision making |
| 8.Satisfaction with treatment decision | PT 101, 105, 106, 108 | - ‘For me, personally, it was sort of just what I had to do [conservative] because I would have to go back to work’ 108 - ‘I feel happy. It’s a really difficult one because given the situation, its like sort of not the best of a bad pick but the position I’ve put myself in with this injury is like not a fantastic one so you either have 2 routes’ 101 - ‘I didn’t walk away, thinking I really feel like I want to go for the surgery and they kind of made me go for the physio, 101’ - ‘I think we were both pretty happy with, like when I walked out of there I was pretty hopeful’ 104 - ‘you’re happy with that decision you’ve chosen so far? P-Yep 100%’ 105 - ‘To be honest I thought because it said physio I thought Id turn up and he’d just rug me through a few movements to try and help it or to help me, I don’t know I didn’t think I was going to get as good of an explanation as I received’ 108 | Whatever the lead up to that point, pts were happy with their decision, whether they made it themselves, shared the responsibility or were told what to do | Theme 3 -Experience of shared decision making |

Themes

1. Understanding of the injury:
   1. Understanding of the injury
2. Factors influencing treatment decision:
   1. Factors influencing treatment decision
   2. Patient expectations
   3. Given multiple options to choose from
3. Experience of shared decision making:
   1. Encouraged to go for a particular option
   2. Patient experiencing decisional uncertainty
   3. Perceived lack of support to make treatment decision
   4. Satisfaction with treatment decision
